# Preferences of Treatment Strategies among Women with Low-Risk DCIS and Oncologists

**DOI:** 10.3390/cancers13163962

**Published:** 2021-08-06

**Authors:** Danalyn Byng, Valesca P. Retèl, Ellen G. Engelhardt, Catharina G. M. Groothuis-Oudshoorn, Janine A. van Til, Renée S. J. M. Schmitz, Frederieke van Duijnhoven, Jelle Wesseling, Eveline Bleiker, Wim H. van Harten

**Affiliations:** 1 Division of Psychosocial Research and Epidemiology, The Netherlands Cancer Institute-Antoni van Leeuwenhoek Hospital, 1066 CX Amsterdam, The Netherlands; v.retel@nki.nl (V.P.R.); e.engelhardt@nki.nl (E.G.E.); e.bleiker@nki.nl (E.B.); w.h.vanharten@utwente.nl (W.H.v.H.); 2 Department of Health Technology and Services Research, Technical Medical Centre, University of Twente, 7522 NB Enschede, The Netherlands; c.g.m.oudshoorn@utwente.nl (C.G.M.G.-O.); j.a.vantil@utwente.nl (J.A.v.T.); 3 Division of Molecular Pathology, The Netherlands Cancer Institute-Antoni van Leeuwenhoek Hospital, 1066 CX Amsterdam, The Netherlands; r.schmitz@nki.nl (R.S.J.M.S.); j.wesseling@nki.nl (J.W.); 4 Division of Surgical Oncology, The Netherlands Cancer Institute-Antoni van Leeuwenhoek Hospital, 1066 CX Amsterdam, The Netherlands; f.v.duijnhoven@nki.nl; 5 Department of Pathology, Leiden University Medical Center, 2333 ZA Leiden, The Netherlands; 6 Department of Clinical Genetics, Leiden University Medical Center, 2333 ZA Leiden, The Netherlands

**Keywords:** active surveillance, de-escalating, discrete choice experiment, ductal carcinoma in situ, patient preference

## Abstract

**Simple Summary:**

Preferences for treatment strategies for low-risk ductal carcinoma in situ (DCIS), a potential precursor of invasive breast cancer (IBC) including a new active surveillance strategy, were elicited with a discrete choice experiment among recently-diagnosed women and oncologists involved in the care of women with DCIS. Patients exhibited strong preferences for active surveillance and seemed prepared to accept much higher levels of 10-year risk of developing ipsilateral invasive breast cancer than oncologists. Both patients and oncologists showed a strong aversion toward more extensive locoregional treatments (i.e., breast conserving surgery followed by radiotherapy, and mastectomy), while both groups demonstrated a strong preference toward shorter follow-up intervals.

**Abstract:**

As ongoing trials study the safety of an active surveillance strategy for low-risk ductal carcinoma in situ (DCIS), there is a need to explain why particular choices regarding treatment strategies are made by eligible women as well as their oncologists, what factors enter the decision process, and how much each factor affects their choice. To measure preferences for treatment and surveillance strategies, women with newly-diagnosed, primary low-risk DCIS enrolled in the Dutch CONTROL DCIS Registration and LORD trial, and oncologists participating in the Dutch Health Professionals Study were invited to complete a discrete choice experiment (DCE). The relative importance of treatment strategy-related attributes (locoregional intervention, 10-year risk of ipsilateral invasive breast cancer (iIBC), and follow-up interval) were discerned using conditional logit models. A total of *n* = 172 patients and *n* = 30 oncologists completed the DCE. Patient respondents had very strong preferences for an active surveillance strategy with no surgery, irrespective of the 10-year risk of iIBC. Extensiveness of the locoregional treatment was consistently shown to be an important factor for patients and oncologists in deciding upon treatment strategies. Risk of iIBC was least important to patients and most important to oncologists. There was a stronger inclination toward a twice-yearly follow-up for both groups compared to annual follow-up.

## 1. Introduction

An active surveillance strategy has been proposed as a new treatment strategy for women with grade I or II primary ductal carcinoma in situ (DCIS), considered a potential precursor of invasive breast cancer (IBC). Between 2014 and 2017, three international, multicenter prospective randomized controlled trials (RCT) evaluating the safety and feasibility of an active surveillance strategy as an alternative to surgical intervention for low-risk DCIS began. Women recruited to the LORD trial in the Netherlands (NCT02492607) [1], the LORIS trial in the United Kingdom (NCT02766881) [2,3], and the COMET trial in the United States (NCT02926911) [4,5] are allocated evenly between the active surveillance arm, and the surgical intervention arm. Women in both arms are followed in the same fashion, with annual mammography (bi-annual in COMET) for a period of up to 10-years post-diagnosis, with ipsilateral invasive breast cancer (iIBC)-free rate as the primary endpoint. All trials have a non-inferiority design, which specifies a clinically meaningful margin for which active surveillance can be considered safe, in terms of the iIBC-free rate, compared to surgical intervention.

Enrolment into these trials was difficult due to strong treatment preferences among eligible woman. Despite public awareness and communication workshops to tackle informational asymmetries in the target population and improve enrolment into the LORIS trial, by the date of the study’s closing in March 2020, only 181 of the targeted 932 women were recruited [6]. Women eligible for the LORD trial demonstrated strong treatment preferences, declining enrolment when randomized to their non-preferred arm. This phenomenon is widely reported for trials with randomization; a systematic review and meta-analysis of partially randomized patient preference trials revealed that more than 50% of refusal of randomization was due to patient preference [7]. This challenge to recruitment may be especially true when no novel treatment option is being offered that potentially improves survival such as in the context of de-escalation trials. If active surveillance is the novel strategy, eligible patients can always de-escalate their own treatment on their own accord and in agreement with their treating oncologists. It is not necessary to enroll into a trial to gain access to the desired treatment and follow-up strategy, unless selecting a de-escalation strategy is informed by risk-stratification using biomarkers not available outside a trial.

The active surveillance trials for DCIS are part of a growing trend toward de-escalation of locoregional and systemic treatment for early breast cancer and DCIS [8]. Given the context of already excellent long-term survival for treated DCIS, numerous studies have evaluated the role and added benefit of radiotherapy and endocrine therapy following surgery [9,10,11,12,13,14,15]. While all studies have reported a reduction of local recurrence following use of radiotherapy or adjuvant endocrine therapy, not one has demonstrated survival benefits from these treatments. Unlike the ongoing active surveillance trials, these previous studies have focused on DCIS without differentiating groups by future risk of iIBC, with the exception of the randomized RTOG 9804 trial. This is the only trial that was restricted to low-risk DCIS, defined by lesion size ≤ 2.5 cm, low or intermediate grade, and negative margins ≥ 3 mm, and aimed to estimate the effect of omitting radiotherapy [10,16]. This trial was open for recruitment between July 1996 and July 2006 and was closed with less than 40% of the originally planned 1790 women accrued.

To tackle difficulties recruiting women, the LORD trial changed from a RCT design to a preference-based design in July 2020. In the COMET trial, the study design allows for ‘crossover’ if a patient randomized to one arm opts for the other (e.g., if a patient randomized to active surveillance opts for surgery in the absence of invasive breast cancer, or vice versa). Rate of crossover is included as a study endpoint [4].

Within the context of low-risk DCIS and apart from trial enrollment, there is a need to explain why particular treatment choices are made and what factors enter into the decision process to better inform shared decision-making processes between patients and physicians. Furthermore, if an active surveillance strategy is deemed safe and effective based on the findings of these studies in the future, incorporating the patients’ preferences in treatment decision making will serve to improve treatment compliance and satisfaction. A woman’s preference for treatment strategy may not only be informed by the extensiveness of the procedure itself, but also what happens afterward: the follow-up regimen and possible outcomes including risk of progression of disease or other impacts on self-image.

In light of the challenges posed by strong preferences that women with recently-diagnosed low-risk DCIS seem to have regarding their treatment strategy, a discrete choice experiment (DCE) was designed to discern their preferences for treatment and follow-up strategies, while capturing the relative importance of treatment characteristics and the acceptable trade-offs that they make between them. An active surveillance strategy may only be deemed acceptable to bring into clinical practice if it can be demonstrated in prospective trials that low-risk DCIS can safely be monitored without causing excess iIBC rates compared to conventional treatment. Therefore, part of this aim was to measure how women weigh the importance of risk of iIBC, relative to other aspects of a treatment strategy. By having health care professionals involved in the care of these women also complete the DCE, a comparison of preferences can also be made between patients and oncologists.

## 2. Materials and Methods

### 2.1. Study Population: Patients and Oncologists

Between June 2019 and June 2020, women with low-risk DCIS who declined enrolment into the LORD trial due to strong preferences for either study arm were invited to participate in the Dutch prospective CONTROL DCIS Registration study. As part of the study, participating women completed a baseline questionnaire including DCE within one month of their enrollment, before possible active treatment. In January 2021, the CONTROL DCIS Registration study was subsequently closed because the LORD trial initially randomizing between active surveillance and conventional treatment was amended to a patient-preference design, similar to the CONTROL study. At closing, 28 (78%) of the women registered to the CONTROL study had selected an active surveillance strategy instead of surgical intervention. From July 2020 onward, newly recruited women to the amended LORD trial have been invited to complete the new version of the baseline questionnaire including the same DCE used for the CONTROL DCIS Registration. Women with completed baseline questionnaires up to February 2021 were included in this analysis.

All eligible women were over 45 years old, diagnosed with primary low or intermediate grade DCIS detected on screening mammography, residing in the Netherlands. At the time of completion of the questionnaire, the women would have chosen either an active surveillance strategy or surgical intervention, but may not yet have undergone the full procedure in the latter option at the time of the baseline questionnaire. The patient respondents included in this study were recruited from 30 hospitals across the Netherlands.

Between October 2019 and December 2020, health care professionals involved in the care of women with DCIS in the Netherlands were invited to participate in the online Health Care Professionals Study questionnaire.

### 2.2. Questionnaire Design for Patients and Health Care Professionals

Patients completing the baseline questionnaire of the CONTROL DCIS Registration and LORD trial (preference-based design) were offered a paper or digital copy of the questionnaire (in Dutch) that comprises questions about socio-demographic characteristics, DCE questions, and health-related quality of life (HRQoL) items. An information letter was also included, outlining the purpose of the study and procedures.

Health care professionals involved in the LORD trial received a personal invitation email to participate in the online Health Professionals Study, and to recruit those not involved in the LORD trial, an email invitation was distributed via the Dutch society for surgical oncology, the Dutch society for radiation oncology and all regional breast cancer working groups affiliated with the Netherlands Comprehensive Cancer Organization. The questionnaire investigated the participants’ own preference for treatment, the impact of clinical characteristics on treatment preference, and need for decision support tools. Surgical oncologists and radiation oncologists completing the online questionnaire were invited to complete the same DCE as the patients.

Approval for the CONTROL DCIS study and Health Care Professionals study was obtained from the Netherlands Cancer Institute’s institutional review board. The Medical Ethics Committee of the Netherlands Cancer Institute approved the LORD patient preference trial.

### 2.3. Intolerance of Uncertainty

In the questionnaire presented to patients, a series of socio-demographic and health-related quality of life (HRQoL) items were included. The responses to the Dutch version of the 12-item Intolerance of Uncertainty Scale (IUS-12) were used for this analysis [17,18]. The IUS-12 assesses self-reported responses to uncertainty, ambiguous situations, and future events. Twelve items are rated on a 5-point Likert scale ranging from 1 (not at all characteristic of me) to 5 (entirely characteristic of me), summing a total score with a maximum of 60 (higher scores indicate greater uncertainty). Intolerance of uncertainty represents a predisposition toward overestimating the chance of possible (but unlikely) undesirable outcomes in uncertain circumstances, while also finding this chance threatening [19]. A threshold to demarcate women with “low” and “high” uncertainty based on the total score was set at the median value resulting from the respondent population in order to compare responses between women with “low” and “high” uncertainty.

### 2.4. Design of the Discrete Choice Experiment

DCEs provide a format to elicit choices in a structured way, making it possible to statistically model binary (“either-or”) choices. They are one of many stated-preference methods in which respondents choose between alternatives in a repeated series of choice tasks. Within each choice, a selection of attributes (e.g., features of the treatment) where varying possible “levels” are provided. The DCE uses an experimental design that determines the presentation of specific attribute-level combinations, out of many possible combinations. This makes it possible to compute the influence of changes in attribute-levels on choice for a treatment strategy [20].

Attributes and their associated levels that capture relevant features of treatment strategies were identified through a review of the literature and expert elicitation. Experts (psychosocial oncology experts (EB, EGE); oncology nurse (VS); pathologist (JW); DCE experts (CGMGO, JAVT)) were asked to comment on and complete the list of attributes and possible levels. The final selection was confirmed via a series of interviews with health care professionals [21]. Treatment attributes included locoregional treatment (levels: no surgery, breast conserving surgery ± radiotherapy, mastectomy), interval between follow-up mammography screening appointments (levels: two years, one year, six months), and chance of ipsilateral invasive breast cancer at 10 years (levels: 5%, 10%, 15%) (Table 1).

For each DCE question (i.e., “choice task”), respondents would choose between two hypothetical treatment strategy alternatives (“Option 1” and “Option 2”) that consist of a unique combination of different attribute levels, determined through an experimental design. All participants were provided educational content on the purpose of the DCE, emphasizing that the treatment strategies and outcomes presented were hypothetical situations. The combination of the strategy alternatives, attributes, and their levels resulted in 90 hypothetical scenarios, derived from a fractional main effects experimental design. Unrealistic combinations of different attribute levels were removed from the experimental design. Presenting all scenarios to respondents would be too burdensome, so a subset of scenarios was used. The R package AlgDesign version 1.2.0 was used to generate a D-efficient design consisting of 36 hypothetical scenarios, divided into three versions of the DCE consisting of 12 choice tasks each. Participants were randomized to receive one of the three versions. An example of a choice task has been provided in the online Supplementary Materials (Methods S1); design restrictions are described in Methods S2.

The minimum required sample size was determined to be *n* = 84, based on the rule of thumb for conditional logit models proposed by Johnson and Orme, taking into consideration the number of choice tasks, alternatives, and analysis cells [22,23].

### 2.5. Conditional Logit Model and Comparing Patient and Oncologist Preferences

To estimate the relative importance of treatment-related features across all respondents, separate conditional logit models for binary choice were built for patients and oncologists. This technique is informed by random utility theory, where a regression model is used to relate choice (i.e., choice of treatment strategy) as a function of the features of the choice (i.e., the attributes and respective levels) [24]. The attributes locoregional treatment, follow-up interval, and risk of subsequent iIBC were included in the model as covariates using dummy coding. Resulting co-efficients (β) were exponentialized to derive odds ratios. *p*-values < 0.05 are considered as statistically significant. In a model with pooled data, an interaction term for respondent type was included for all dummy-coded attribute-levels to determine where preferences differed between patients and oncologists. To test the significance of the overall interaction between different attributes and respondent type, the likelihood ratio test was conducted comparing models with and without the interaction terms, with two degrees of freedom.

To understand the relative contribution of the attribute-level to the utility that the respondent assigns to an alternative, importance weights were calculated separately for patients and oncologists. Utility can be understood as the measure of value or importance, and consequently important weights represent the relative importance of each level. These importance weights are the resulting coefficients (β) of the conditional logit models. The overall importance weight (*OIW*) of each attribute (i) was calculated by dividing the range in regression coefficients of each attribute i (i.e., the difference between the least and most preferred attribute levels, $maxC_{i}-minC_{i}$), by the sum of the coefficient ranges of the three attributes ($maxC_{j}-minC_{j}$).

(1) $$OIW_{Attribute{\;}_{i}}=\frac{maxC_{i}-minC_{i}}{\Sigma_{k}\left(maxC_{j}-minC_{j}\right)}$$

Scaled overall importance weights (as a fraction of 100) were then derived for each attribute, together summing 100. Overall importance weights were calculated separately for oncologists, patients, and for patient subgroups (women with “high” and “low” uncertainty intolerance, as determined by the IUS-12 cohort median value, patients undergoing active surveillance, patients undergoing conventional treatment, and women with “high” and “low/intermediate” educational attainment). A description of an effect-modifier analysis to study the extent to which certain patient characteristics impacted the preferences of respondents is described in the online Supplementary Materials (Methods S3).

Maximum acceptable risk was calculated for patients based on the resulting coefficients from the conditional logit model. This can be understood as what extra risk of ipsilateral invasive breast cancer at 10 years patients are willing to take for getting no treatment compared to breast conserving surgery. This is calculated by dividing coefficients to determine the change in risk of ipsilateral invasive breast cancer that would offset the utility gain of the most preferred locoregional treatment strategy.

All statistical analyses were performed with R version 3.6.1 (R Foundation for Statistical Computing, Vienna, Austria). The R package mlogit version 1.1-1 was used for the conditional logit models.

## 3. Results

### 3.1. Respondents

A total of 202 individuals completed the questionnaire including DCE by March 2021; 37 patients from the CONTROL DCIS registration, 135 patients from the LORD trial, and 30 radiation and surgical oncologists from the Health Professionals Study (Table 2). Patients had a mean age of 59 (range 45–77), and 95 (55.2%) were engaged in paid labor (part-time or full-time). A total of 76.7% opted for no surgical intervention for their primary low-risk DCIS. Responses on the IUS-12 uncertainty intolerance scale ranged between 20 and 51, with the median value at 30. A total of 70% of the oncologist respondents were female. More than 50% treated more than 15 women with DCIS per year. Twenty oncologists (67%) specialized in surgical oncology and the remaining in radiation oncology; all were employed at a range of hospitals across the Netherlands (Table 2).

### 3.2. Importance of Treatment Characteristics

**Figure 1:**
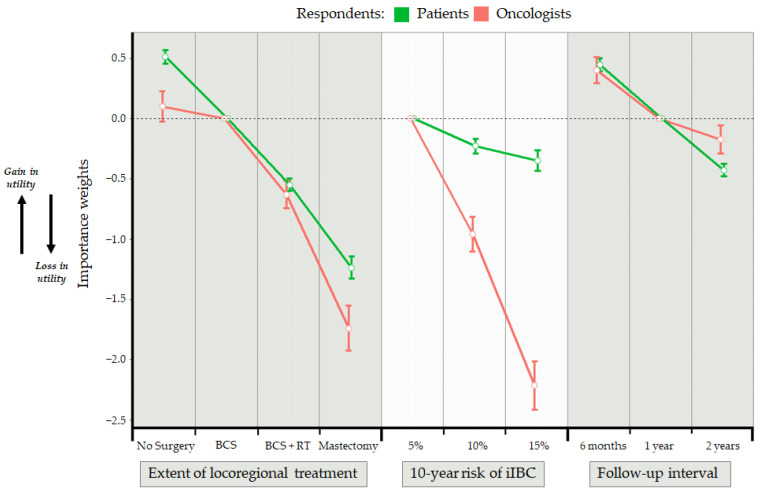
Importance weights derived from the conditional logit model. Standard error bars shown are an indication uncertainty in respondents’ preferences. Importance weights for each attribute-level shown are a measure of relative preference. Moving from one attribute-level to an adjacent attribute-level is an indication of the relative gain or loss in utility, where utility is a representation of the strength of preferences. BCS: breast conserving surgery; iIBC: ipsilateral invasive breast cancer; RT: radiotherapy.

Table 3 shows the aggregate results of the discrete choice experiment for patients and oncologists separately based on the conditional logit models. Model coefficients are also plotted in Figure 1. The preferred locoregional treatment option for patients and oncologists was no surgery, then breast conserving surgery, followed by breast conserving surgery and radiotherapy. The least preferred option was mastectomy. A follow-up interval of six months was preferred by all respondents. Patients did not assign large relative importance to any of the possible levels of iIBC risk whereas for oncologists, iIBC risk was a very important factor. There was a statistically significant difference between the oncologists and patients in their preference of attributes (likelihood ratio test on interaction between respondent type and attribute, *p* < 0.001) (Table 3). The test of interaction between respondent type and the attribute-level 15% risk of iIBC was also statistically significant (*p* = 0.02). Oncologists and patients were not statistically significantly different in their preference for the other attribute-levels for follow-up interval or locoregional treatment.

We determined what extra risk of ipsilateral invasive breast cancer at 10 years patients were willing to take for getting no surgery compared to breast conserving surgery. This calculation of maximum acceptable risk found that the additional increase in risk (from the reference level of 5%) that exactly offsets the increase in utility of having no surgery (i.e., not experiencing the side-effects) would be 11.2%.

### 3.3. Influence of the Attributes on Patients’ and Oncologists’ Preference

**Figure 2:**
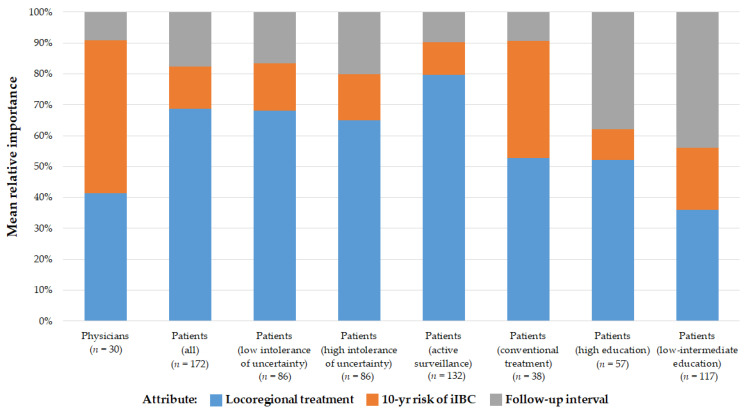
Overall relative importance weights for attributes (features) of treatment strategy. Exploration of further patient subgroup stratifications are described in the online Supplementary Materials (Methods S3). iIBC: ipsilateral invasive breast cancer.

Scaled overall importance weights for each attribute are shown in Figure 2. These weights represent the relative influence of an attribute on the respondents’ preference for a treatment strategy. For patients, 10-year risk of iIBC was the least important attribute dictating preference, whereas this was the most important for oncologists (representing 14% vs. 50% of importance). For patients, the locoregional treatment was the most important attribute dictating preference, followed by follow-up interval. Heterogeneity in preferences exists among patient subgroups. Women who chose a conventional treatment strategy in real life assigned a higher overall importance weight to 10-year risk of iIBC compared to women who chose active surveillance (38% vs. 11%). Models were built separately for women split by their scores in the bottom and top half of the intolerance of uncertainty scale. Women with higher uncertainty intolerance scores seemed to attach higher importance to follow-up interval slightly more than their counterparts on the other side of the scale (20% vs. 17%). The relative importance of iIBC risk was the same for both groups. When inspecting relative importance of attributes by education level, the importance of locoregional treatment and follow-up interval was shown to be nearly equal.

## 4. Discussion

The extensiveness of the locoregional treatment was consistently shown to be an important factor for patients and their care providers in deciding upon treatment strategies for low-risk primary DCIS. In our analyses, risk of ipsilateral invasive breast cancer was least important to patients and most important to oncologists. There was a stronger inclination toward a twice-yearly follow-up for both oncologists and patients compared to annual follow-up.

We found that women in the Netherlands had very strong preferences for an active surveillance strategy with no surgery, irrespective of the 10-year risk of iIBC. This was also the case for our respondents who scored higher than the cohort’s average score (30) on the IUS-12 uncertainty intolerance scale. These women, known to have a higher intolerance of uncertainty, were not dissimilar to their counterparts with lower intolerance in assigning a comparatively small overall importance weight to the risk of iIBC. For these women, the locoregional treatment, followed by the interval between follow-up mammograms, were more important. It is possible that the IUS-12 uncertainty intolerance scale does not capture future breast cancer risk tolerance in comparison to tolerance of risk attributed to other attributes (e.g., risk of infection or post-operative complications) [25,26]. Furthermore, the risk of iIBC already remains rather low among these women with good-prognosis DCIS, and they are being asked to evaluate a risk far in the future at 10 years. The women in our study not only attached lower importance to future risk of breast cancer, but also attached higher importance to breast conservation through having no surgery. This can be aligned with prospect theory, popularized by Kahneman and Tversky, which posits that “people underweigh outcomes that are merely probable in comparison with outcomes that are obtained with certainty” [27]. It is not yet understood how the dimension of temporal distance to the risk in question factors into decision making and preferences measured in DCEs, particularly for DCIS [28]. A study of intolerance of uncertainty among men undergoing active surveillance for prostate cancer found that intolerance of uncertainty had a significant relationship with the experience of cancer-related symptoms [29]. The women in our study were asymptomatic and their DCIS was detected through the national breast cancer screening program, so they remain physically unaffected by their diagnosis.

An important related consideration that likely factors into a patient’s choice is the understanding of one’s personal risk of upstaging to invasive breast cancer; this was not explicitly captured in the DCE design. Uncertainty still remains over the proportion of patients with a core needle biopsy showing DCIS with “low-risk” clinicopathological characteristics who actually have concurrent invasive carcinoma in the breast. This uncertainty is now understood to have an impact on participation in trials studying active surveillance. A retrospective series based on a small sample of women who would have met eligibility criteria for active surveillance trials found low upstaging rates (6–10%) [30]. All upstaged cases were good-prognosis invasive carcinomas: all were node negative and HER2 negative. Furthermore, a proportion of women with DCIS will have complete removal of the lesion at biopsy, and subsequently experience a low upgrade rate (8.2%) [31]. A study in the Netherlands addressed the issue of the reliability of preoperative biopsy, and identified several factors that can aid in further risk stratification of women being considered for non-operative management [32]. An important takeaway from these studies is that even with possible upstaging, overall survival should not be significantly compromised. Access to high-quality annual mammography is readily available, and invasive carcinomas can be treated on time. Nevertheless, the prediction of upstaging of DCIS to invasive disease remains an important area of ongoing research, and will serve to identify the lowest achievable upstaging rate among women eligible for clinical trials of active surveillance. This may in turn address some of the challenges with trial accrual, and better inform the understanding of risk of upstaging.

We used a discrete choice experiment as a “stated preference” method where respondents were asked to choose between alternatives from among a set of hypothetical scenarios generated from an experimental design [33]. This can be contrasted with the concept of “revealed preference” in which we observe actual choices made by respondents in real life. The women included in our study were participants in studies (the CONTROL DCIS Registration and LORD trial) that had a preference-based design. Sixty-eight percent of our patient respondents selected active surveillance as an alternative to surgical intervention in real life. Active surveillance is not yet an accepted treatment strategy according to European clinical guidelines. Conventional treatment for DCIS mimics that of early breast cancer, with breast conserving surgery being the preferred local treatment option [34]. Results from the ongoing prospective clinical trials for active surveillance will not be available for at least five to 10 years, and recruitment into these trials remains challenging. However, in the Dutch context, our study demonstrated that women diagnosed with low- and intermediate-grade DCIS have already established strong preference and desire to undergo active surveillance ahead of the results about safety and 10-year risk of ipsilateral invasive breast cancer.

The non-inferiority design of the Dutch LORD trial is based on the assumption that the 10-year iIBC-free rate is 95% in the surgery group. The non-inferiority margin was chosen at 3.168 on the hazard-ratio scale, corresponding to a 10-year iiBC-free probability of 85% in the active surveillance group [35]. As DCE scenarios presenting active surveillance were always associated with an increased risk of 5% or 10% compared to the surgical treatment option, we found that these differences were deemed acceptable by the patient respondents. Even when two surgical treatment options were compared, patients had much stronger preferences for strategies with less extensive procedures, irrespective of an associated increased risk. This pattern was not seen among oncologist respondents; a difference of 10% in risk was not deemed acceptable by oncologists on average.

This DCE is the first published study evaluating treatment preferences in women with a recent diagnosis of DCIS. A DCE evaluating patient preferences for outcomes following DCIS treatment was conducted in a healthy cohort of women in the United States attending a comprehensive cancer screening mammography clinic [36]. These women were not diagnosed with DCIS, nor did they have a personal history of breast cancer. That study found that respondents weighed breast cancer risk as the most important factor, but this was closely followed by chronic pain and infection. Again, this is in contrast with the patient respondents in our study who demonstrated that 10-year iIBC risk was the least important factor. The extent to which women without the experience of the disease in question respond similarly to women with the disease is known to be affected by scale heterogeneity, explained by differences between groups due to familiarity with the disease [37]. In the online Supplementary Materials (Methods S4, Figure S1), we provide an evaluation of scale heterogeneity between the patients and oncologists in our sample to understand how similarly these groups respond. We also note that differences in sample size between patients and oncologists may have impacted the difference observed between these two groups. These considerations are necessary to draw comparisons between preferences of any two groups of individuals including women who have been diagnosed with DCIS and those not, to understand the influence of psychological distance on accepting treatment strategies with possible higher risk of a future iIBC event [38].

## 5. Conclusions

This study provided insights into the treatment strategy preferences of a large cohort of women participating in a preference-based prospective study for low-risk DCIS. These women, recently diagnosed with DCIS, assigned the greatest importance to extensiveness of locoregional treatment and surveillance follow-up interval. In stark contrast, risk of iIBC was the most important factor for oncologists involved in the care of DCIS. The responses to the DCEs are reflected in the women’s actual treatment choices: the vast majority (68%) chose an active surveillance strategy to manage their low-risk DCIS. The insights gained through this study about the concordant and discordant preferences for treatment strategies between women and their oncologist may help to inform treatment decision making processes as prospective trials aim to recruit more women. Finally, if an active surveillance strategy is found to be a safe alternative to surgery, incorporating patients’ preferences in treatment decision making will serve to improve strategy compliance, satisfaction, and shared-decision making processes.

## Figures and Tables

**Table 1:** Attributes and their respective levels in the discrete choice experiment.

Attributes	Levels
Locoregional treatment strategy	No surgery; breast conserving surgery; breast conserving surgery followed by radiotherapy; mastectomy
10-year risk of ipsilateral invasive breast cancer (iIBC)	5%; 10%; 15%
Surveillance mammography follow-up interval	6 months; 1 year; 2 years

**Table 2:** Patient and oncologist characteristics.

Characteristics	Patients (*n* = 172) *N* (%)	Oncologists (*n* = 30) *N* (%)
Age, years (median, range)	59 (45–77)	N.A.
Sex		
Female	172 (100%)	21 (70.0%)
Male	0	9 (30.0%)
Actual treatment selected		
Active surveillance	132 (76.7%)	N.A.
Conventional treatment	38 (22.1%)	N.A.
Unknown	2 (1.2%)	N.A.
Educational level		
Low	37 (21.5%)	0
Intermediate	78 (45.3%)	0
High	57 (33.1%)	30 (100%)
Employment status		
Employed (part-time or full-time)	95 (55.2%)	30 (100%)
Unemployed/pension	77 (44.8%)	0
Hospital type		
Academic medical center	3 (1.7%)	8 (26.7%)
General teaching hospital	105 (61.0%)	14 (46.7%)
Specialized oncology hospital	25 (14.5%)	5 (16.7%)
General hospital	39 (22.7%)	3 (10.0%)
Region of the Netherlands		
North	3 (1.7%)	3 (10.0%)
East	60 (34.9%)	5 (16.7%)
West	98 (57.0%)	17 (56.7%)
South	11 (6.4%)	5 (16.7%)
Subspecialty		
Surgical oncology	N.A.	20 (66.7%)
Radiation oncology	N.A.	10 (33.3%)
Number of patients with DCIS treated per year		
2–5 patients	N.A.	1 (3.3%)
6–10 patients	N.A.	7 (23.3%)
11–15 patients	N.A.	3 (10.0%)
16–20 patients	N.A.	11 (36.7%)
>20 patients	N.A.	8 (26.7%)
Years’ experience treating patients with DCIS		
2–5 years	N.A.	1 (3.3%)
6–10 years	N.A.	9 (30.0%)
>10 years	N.A.	20 (66.7%)

**Table 3:** Stated preferences across all respondents based on the conditional logit model with dummy coding.

Attribute Levels	Patients			Oncologists		
	Coefficient (β)	SE	Exp (β)	Coefficient (β)	SE	Exp (β)
Locoregional treatment						
Breast conserving surgery	(ref.)			(ref.)		
No surgery	0.513 *	0.111	1.67	0.100	0.251	1.11
Breast conserving surgery + radiotherapy	−0.551 *	0.102	0.58	−0.632 *	0.229	0.53
Mastectomy	−1.239 *	0.185	0.29	−1.743 *	0.371	0.18
10-year risk of ipsilateral invasive breast cancer						
5%	(ref.)			(ref.)		
10%	−0.229	0.122	0.79	−0.962 *	0.290	0.38
15%	−0.350 *	0.174	0.70	−2.219 *	0.399	0.11
Interval surveillance follow-up						
1 year	(ref.)			(ref.)		
6 months	0.448 *	0.110	1.56	0.403	0.218	1.50
2 years	−0.429 *	0.103	0.65	−0.175	0.235	0.84
**Interaction Terms ^a^**			**Coefficient (β)**	**SE**	**Exp (β)**	***p*-Value**
Attribute: Locoregional treatment * respondent type						<0.001 ^b^
Level: No surgery						
Patient			(ref.)			
Oncologist			−0.413	0.275	0.66	0.13
Level: Breast conserving surgery + radiotherapy						
Patient			(ref.)			
Oncologist			−0.082	0.251	0.92	0.75
Level: Mastectomy						
Patient			(ref.)			
Oncologist			−0.504	0.414	0.60	0.22
Attribute: 10-year risk of ipsilateral invasive breast cancer * respondent type						<0.001 ^b^
Level: 10% risk of iIBC						
Patient			(ref.)			
Oncologist			−0.734	0.314	0.48	0.02
Level: 15% risk of iIBC						
Patient			(ref.)			
Oncologist			−1.870	0.435	0.15	<0.001
Attribute: follow-up interval * respondent type						<0.001 ^b^
Level: 6mo follow-up interval						
Patient			(ref.)			
Physician			−0.045	0.245	0.96	0.85
Level: 2 year follow-up interval						
Patient			(ref.)			
Oncologist			0.254	0.258	1.29	0.32

iIBC: ipsilateral invasive breast cancer; ref.: reference level; * Statistically significant *p*-value < 0.05; ^a^ Computed from model using pooled data from all respondents, with interaction term for respondent type. ^b^ Based on the likelihood ratio test comparing models with and without the interaction terms, with 2 degrees of freedom.

## Data Availability

Data may be available on request due to privacy restrictions, from the corresponding author.

## Supplementary Materials

The following are available online at https://www.mdpi.com/article/10.3390/cancers13163962/s1, Methods S1: Example of a choice task, Methods S2: Design restrictions, Methods S3: Assessing patient heterogeneity with effect-modifier analyses, Methods S4: Analysis of the scale factor, Figure S1: Swait and Louviere plot of coefficients derived from conditional logit models for patients and oncologists.
