# Supplementary material for: Preferences of Treatment Strategies among Women with Low-Risk DCIS and Oncologists
Source: Cancers (Basel). 2021 Aug 6;13(16):3962. doi: 10.3390/cancers13163962 (PMC8394332; doi:10.3390/cancers13163962)
Supplement: Supplementary file 1 [file cancers-13-03962-s001.zip › cancers-1315138-supplementary.pdf]

Supplementary Materials

Preferences of Treatment Strategies among Women with Low-Risk DCIS and Oncologists

Danalyn Byng, Valesca P. Retèl, Ellen G. Engelhardt, Catharina G.M. Groothuis-Oudshoorn, Janine A. van Til, Renée S.J.M. Schmitz, Frederieke van Duijnhoven, Jelle Wesseling, Eveline Bleiker and Wim H. van Harten on behalf of the Grand Challenge Precision Consortium

Methods S1: Example of A Choice Task

Characteristics	OPTION A	OPTION B
Extent of the surgical procedure	**Mastectomy** with possible (immediate) reconstruction 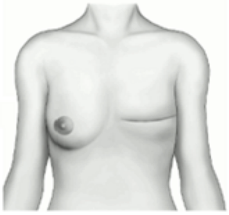 **Moderate** side effects	**No surgery** 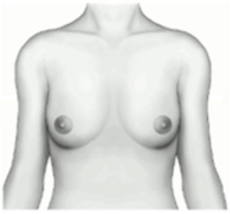 **No** side effects
Extent of the irradiation	No irradiation	No irradiation
Frequency of follow-up appointments	Annual (once yearly)	Biannual (twice yearly)
Chance of developing invasive ipsilateral breast cancer within 10 years following the DCIS diagnosis	**5 in 100 women (5%)**	**15 in 100 women (15%)**
QUESTION: Which treatment option do you prefer?	○ OPTION A	✓ OPTION B

In the experiment, respondents are presented with a series of questions in which they are asked to choose a preferred alternative from a set of hypothetical treatment profiles. These treatment profiles vary by levels of treatment factors, shown here and in Figure 1 of the main manuscript.

Our experimental design resulted in 36 choice tasks, and we divided these into three blocks. Each individual was randomized to complete one of the three blocks, containing 12 choice tasks. One example choice task is shown above.

Methods S2: Design Restrictions

To ensure the presentation of hypothetical scenarios with a closer representation of what would be seen in the real-world, this study used restrictions in the DCE design. In the presentation of treatment strategies, the option with a more invasive local intervention was always associated with a lower chance of iIBC at 10 years. Choice tasks always compared different locoregional treatments; as such, the "no surgery" level of the locoregional treatment attribute was never associated with the "5%" level of the risk of iIBC attribute.

Methods S3: Assessing Patient Heterogeneity with Effect-Modifier Analyses

A series of multivariable mixed logit models were built including responses from patients only. To study the extent to which certain patient characteristics impacted the stated preferences of respondents, an effect-modifier analysis was conducted by including interaction terms for age, highest level of education completed, and employment status. Nested random effects for respondent ID and hospital were included to account for correlation among the multiple questions answered per individual, as well as the possible correlation for respondents treated in the same hospital. We found no statistically significant interactions for age (< vs >50 years) or employment status. A multivariable mixed logit with random effects only for respondent ID included an interaction term for hospital type ("Specialized oncology hospital, Y/N"). This was also not statistically significant.

We did however find that compared to women with low/intermediate educational attainment, women with a high level of education demonstrated aversion to mastectomy (coefficient −0.38, p value 0.04), and preference towards breast conserving surgery with radiotherapy (coefficient 0.27, p value 0.004), compared to breast conserving surgery alone. Overall importance weights stratified by high and low/intermediate educational attainment are shown in Figure 2.

Methods S4: Analysis of the Scale Factor

The assumption of homogeneous utility weights requires that unobservable components of utilities should be mutually independent and homoscedastic [1]. The potential for preference and scale heterogeneity in responses for the total sample and by subgroups (i.e., oncologists and patients) should be therefore measured and accounted for. As it is likely that variances differ between datasets derived from women with DCIS and oncologists, attribute-level estimates (i.e., preferences) between both groups cannot be directly compared without first considering scale factor differences using the Swait and Louviere test [2]. Preference differences between patients and oncologists may differ due to real difference in preference or due to scaling. The latter comes from more or less certainty one has over their preferences, in other words, the scale factor can be understood as a measure of the psychological distance that individuals from different groups (e.g., patients and oncologists) have towards given events. This is due to the perfect confound between the mean and variance of the betas.

To compute the Swait and Louviere test, the log likelihoods derived from the conditional logit models for both groups' datasets were collected (L_1 and L_2). The attribute-level codes for one dataset were multiplied by a possible scale factor, then the two datasets were combined to derive a pooled log likelihood (L_μ). These steps were repeated for a range of possible scale factors, until a log likelihood representing the model with the best fit was found. This is compared with the log likelihoods derived from the separate models from each group, then compared with the chi-square value of the number of parameters in the model (K) plus 1 (representing degrees of freedom), as outlined in the following formula [3,4]:

$$\lambda_A = -2^*\left[L_\mu - (L_1 + L_2)\right] < x^2$$

Following the steps of the Swait & Louviere test, the hypothesis of equal attribute level estimates was rejected ($p < 0.05$). When varying the scale parameter from 0 to infinity, the corresponding maximal log likelihood differed significantly with the sum of the separate log likelihoods of the two models (patients and oncologists). Therefore it can be concluded that irrespective of the value of the scale parameter, there is always a difference between patients and oncologists in their preferences. Due to the contrast in sample sizes between groups, it is important to consider that the results of the Swait & Louviere test may produce different findings with a larger sample of oncologists.

Coefficients from the patient and oncologists models are also plotted in Figure S1 (referred to as the Swait and Louviere plot). Corresponding to the slope of the line fitted through the points the figure suggests that coefficients between the models differ by a scalar of approximately 0.45.

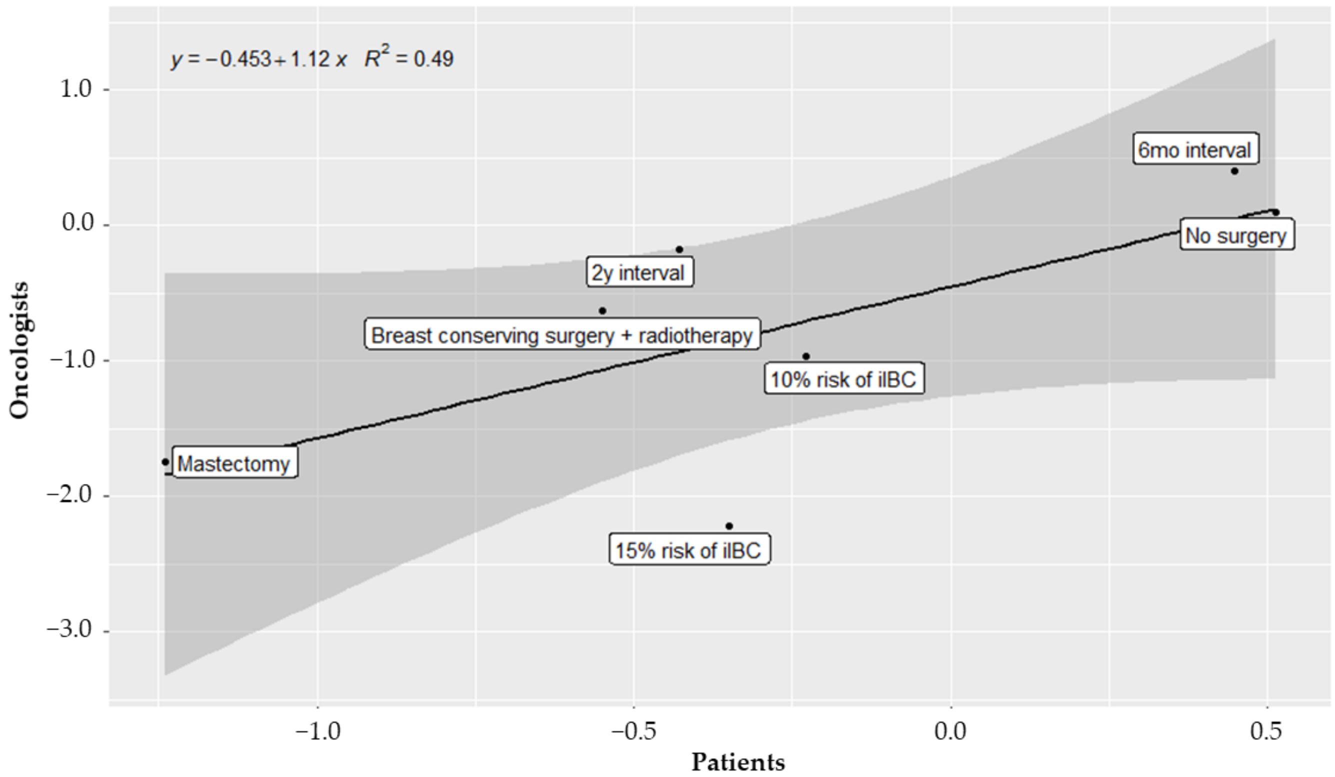

Figure S1. 1 Swait and Louviere plot of coefficients derived from conditional logit models for patients and oncologists. iIBC: ipsilateral invasive breast cancer; mo: month; y: year.

References

1. McFadden, D. Conditional Logit Analysis of Qualitative Choice Behavior. In P. Zarembka (ed.), *Frontiers in Econometrics;* Academic Press: New York: 1973; pp. 105–142.
2. Swait, J.; Louviere, J. The role of the scale parameter in the estimation and comparison of multinomial logit models. *Journal of Marketing Research* **1993**, *30*, 305–314.
3. Vass, C.M.; Wright, S.; Burton, M.; Payne, K. Scale heterogeneity in healthcare discrete choice experiments: A primer. *Patient* **2018**, *11*, 167–173.
4. Veldwijk, J.; Groothuis-Oudshoorn, C.G.M.; Kihlbom, U.; Langenskiöld, S.; Dekker, E.; Kallenberg, F.G.J.; de Wit, G.A.; Lambooij, M.S. How psychological distance of a study sample in discrete choice experiments affects preference measurement: A colorectal cancer screening case study. *Patient Prefer. Adherence* **2019**, *13*, 273–282.
